# Personalized treatment supported by automated quantitative fluid analysis in active neovascular age-related macular degeneration (nAMD)—a phase III, prospective, multicentre, randomized study: design and methods

**DOI:** 10.1038/s41433-022-02154-8

**Published:** 2022-07-05

**Authors:** Leonard M. Coulibaly, Stefan Sacu, Philipp Fuchs, Hrvoje Bogunovic, Georg Faustmann, Christian Unterrainer, Gregor S. Reiter, Ursula Schmidt-Erfurth

**Affiliations:** 1 Vienna Clinical Trial Centre (VTC), Department of Ophthalmology and Optometry, Medical University of Vienna, Vienna, Austria; 2 Christian Doppler Laboratory for Ophthalmic Image Analysis, Department of Ophthalmology and Optometry, Medical University of Vienna, Vienna, Austria; 3 RetInSight, Vienna, Austria

**Keywords:** Macular degeneration, Prognostic markers

## Abstract

**Introduction:**

In neovascular age-related macular degeneration (nAMD) the exact amount of fluid and its location on optical coherence tomography (OCT) have been defined as crucial biomarkers for disease activity and therapeutic decisions. Yet in the absence of quantitative evaluation tools, real-world care outcomes are disappointing. Artificial intelligence (AI) offers a practical option for clinicians to enhance point-of-care management by analysing OCT volumes in a short time. In this protocol we present the prospective implementation of an AI-algorithm providing automated real-time fluid quantifications in a clinical real-world setting.

**Methods:**

This is a prospective, multicentre, randomized (1:1) and double masked phase III clinical trial. Two-hundred-ninety patients with active nAMD will be randomized between a study arm using AI-supported fluid quantifications and another arm using conventional qualitative assessments, i.e. state-of-the-art disease management. The primary outcome is defined as the mean number of injections over 1 year. Change in BCVA is defined as a secondary outcome.

**Discussion:**

Automated measurement of fluid volumes in all retinal compartments such as intraretinal fluid (IRF), and subretinal fluid (SRF) will serve as an objective tool for clinical investigators on which to base retreatment decisions. Compared to qualitative fluid assessment, retreatment decisions will be plausible and less prone to error or large variability. The underlying hypothesis is that fluid should be treated, while residual persistent or stable amounts of fluid may not benefit from further therapy. Reducing injection numbers without diminishing the visual benefit will increase overall patient safety and relieve the burden for healthcare providers.

**Trial-registration:**

EudraCT-Number: 2019-003133-42

## Background

Nearly 196 million people worldwide are estimated to be affected by age-related macular degeneration (AMD). With a growing proportion of the elderly population worldwide, AMD is projected to affect up to 288 million people by 2040 [1]. Optimization of AMD treatment is crucial in the fight against visual impairment and severe, irreversible vision loss. From a clinical point of view AMD can be classified into three stages: early, intermediate and late stage [2]. Neovascular AMD (nAMD), caused by macular neovascularization, rapidly affects central vision and, if left untreated, leads to fibrotic scarring and irreversible loss of visual function [3].

The treatment of nAMD was revolutionized by the introduction of anti-vascular endothelial growth factor (anti-VEGF) leading to a substantial initial restoring of visual acuity (VA) [4]. Anti-VEGF has therefore become the gold-standard treating nAMD [5]. Due to the chronic progressive nature of the disease, frequent retreatments are necessary in order to achieve stabilization of VA. A high frequency of intravitreal injections comes with a recurrent risk for complicated sight-threatening adverse events like endophthalmitis, intraocular inflammations or rhegmatogenous retinal detachment [6]. Furthermore, anti-VEGF administration requires a substantial logistical and financial effort from healthcare systems and caregivers, as treatment visits are accompanied by regular follow-up examinations [7, 8]. Thus, the attempt to minimize the number of retreatments without compromising therapy-benefits. Several different therapy strategies have been proposed to individualize the treatment with anti-VEGF. The objective of these different models is to find a balance between frequent intravitreal injections, disease progression and restoration of VA while minimizing the risk of under- or overtreatment.

Disease activity encouraging retreatment decisions in more individual treatment regimens are determined by vision loss and predefined morphologic disease biomarkers. Spectral-domain optical coherence tomography (SD-OCT) has been established as the leading diagnostic tool in everyday clinical routine to determine disease activity due to its ability to non-invasively visualize the retinal compartments and their respective pathological changes in a three-dimensional manner [9]. Exudative processes, pathognomonic in nAMD, like neovascular leakage comes with related fluid accumulations inside the retina as IRF and/or underneath the retina as SRF or underneath the pigment epithelium as pigment epithelial detachment (PED) which can be observed in OCT-imaging as reliable indicators of disease activity [10]. Still, real-world therapy effectiveness for nAMD has been underwhelming in comparison to the vision gains achieved in clinical trials [11]. To minimize the risk for over- or undertreatment reliable and measurable imaging biomarkers in retreatment decisions need to be established in a precise and reproducible manner [12].

Recent studies found distinct structure-function correlations for specific exudative processes in nAMD. IRF has been consistently associated with severe and irreversible vision loss [13]. The role of SRF seems more ambiguous. Its presence in the juxtafoveal location is associated with negative effects on VA [14], but might be tolerable to some degree without sacrificing therapy benefits [15]. Nonetheless these findings underline the importance of fluid volumes in the pathophysiological process of nAMD and therefore the need for a more precise quantitative assessment of exudative processes. The mere qualitative fluid assessment as present or absent is lacking the important dimension of volumes [15, 16]. Even more so it is prone to error as the CATT and FLUID trials have both shown recurrent differing interpretations of macular fluid between reading centres and ophthalmologists, especially regarding the detection of IRF. Until recently, a precise qualitative fluid assessment was associated with an immense effort by the healthcare system as segmentation of macular fluid was done manually by human graders. With an increasing number of patients, it is unrealistic to manually assess fluid volumes on OCT scan, particularly in a clinical setting. The introduction of AI-algorithms based on deep learning for OCT image analysis are therefore paving the way for a paradigm shift in the treatment of retinal disease [17]. Their ability to analyse large sets of imaging data in a short time will help to minimize diagnostic and therapeutic errors and foster personalized medicine. Additionally, AI can highlight new disease-specific patterns that could strongly deepen our understanding of the pathophysiology in retinal diseases [18]. Combining these innovative technologies with the understanding of the role of exudative processes in nAMD might diminish the gap in treatment outcomes between the real-world and clinical studies.

The here presented prospective randomized controlled trial will be conducted to investigate the implementation of AI-supported macular fluid assessments in a real-world setting, compare AI-supported quantitative fluid measurements to conventional physician-based qualitative OCT assessments and identify the most efficient and relevant biomarker conditions which are providing highest vision gains with the lowest burden of interventions.

### Study objectives

The aim of this study is to compare treatment enriched with automated quantitative fluid volume measurement, with the conventional qualitative fluid assessment in the management of active nAMD. We introduce an optimized pro-re-nata (PRN) regimen, supported by AI fluid quantifications, in a real-world setting, with the goal to increase treatment effectiveness. Effectiveness will be evaluated by the primary outcome, a reduction in the number of retreatments. Secondary outcomes encompass BCVA assessment, morphologic or sensitivity changes in the macular retina, perfusion shifts in the choroid, retina or in neovascular lesions, fibrosis formation and improvements in quality-of-life.

The goal of this optimized AI-supported PRN regimen is to guarantee patient safety and visual outcomes while lessening the treatment burden for the patients, physicians and budget holders alike.

## Methods and design

### Study design and setting

This study will be carried out in accordance with the ICH-GCP and follow the tenets of the declaration of Helsinki including current revisions.

**Fig. 1:**
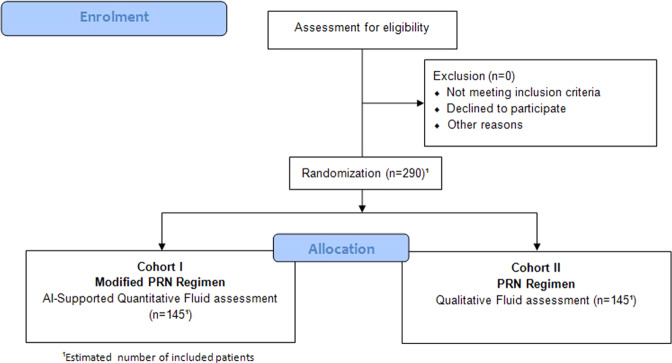
Consort study diagram. Flow chart outlining patient enrolment and randomization.

This phase III clinical trial will be prospective, multi-centred, multinational, randomized (1:1) patient and best-corrected visual acuity (BCVA)-examiner masked. The study aims to recruit two cohorts of 145 patients each, adding up to a total study population of 290 patients. Figure 1 outlines the enrolment and randomization process.

Patient eligibility will be assessed during the baseline (BSL) visit and will follow the predefined inclusion (summarized under Table 1) and exclusion criteria. Recruited patients will be over 50 years of age and have active nAMD defined as presence of foveal intra- and/or subretinal fluid on OCT. BCVA will be measured with the Early Treatment Diabetic Retinopathy Study (EDTRS) charts and must be better or equal to 20/200 Snellen-equivalent at the beginning of the study.

**Table 1:** Key inclusion criteria.

Active nAMD with foveal intra- and/or subretinal fluid
BCVA better or equal to 1.0 log MAR (Snellen equivalent: 20/200)
Patient age ≥ 50 years
Willingness and ability to comply with study visits and study procedures
Signed informed consent form

Eyes of patients with any other concurrent intraocular condition that could, by the discretion of the investigators, interfere with treatment efficacy will not be included in the study. Therefore, eyes suffering from macular holes, diabetic macular oedema, outer retinal atrophy due to other diseases than AMD, late-stage glaucoma (with C/D ratios > 0.9), history of uncontrolled glaucoma (defined as intraocular pressure ≥25 mmHg) under IOP lowering treatment, presence of corneal decompensation, haze or scaring with an impact on BCVA will be excluded from our study population. Further, patients with any surgical treatment of the study eye within the last 3 months prior to baseline will be excluded. Previous anti-VEGF treatment of nAMD will not be considered an exclusion criterion. Therefore, patients with previous anti-VEGF treatment can still be included into the study population, if active nAMD is present.

The recruitment process will take place at different national and international study sites:

(I) Department of Ophthalmology and Optometry, Medical University of Vienna, Vienna, Austria;
(II) Department of Ophthalmology, Landeskrankenhaus Horn, Lower Austria, Austria;
(III) Department of Ophthalmology, Hopital de la Croix, Lyon, France;
(IV) Department of Ophthalmology, Centre Hospitalier Intercommunal, Creteil, France;
(V) Department of Ophthalmology, CHU Montpellier, Hopital Gui de Chauliac, Montpellier, France;
(VI) Department of Ophthalmology, Rhena Clinique de Strasbourg, Strasbourg, France;
(VII) Department of Ophthalmology, CHU Dijon, Dijon, France;
(VIII) Department of Ophthalmology, University Hospital Zurich, Zurich, Switzerland;
(IX) University Eye Clinic, San Giuseppe Hospital, IRCCS MultiMedica, Milan, Italy.

### Study procedures

Patients will be randomized 1:1 into an AI-supported PRN regimen with quantitative fluid volume measurement available for decision-making or a conventional PRN regimen, in which fluid is assessed by the treating physician in a qualitative manner. Patients in both study arms will undergo monthly scheduled visits at their respective study site. During every visit, patients receive a standardized ophthalmologic examination including BCVA tests using ETDRS charts, a slit-lamp biomicroscopy (SL) with fundoscopy (FD) and intraocular pressure (IOP) measurement.

Morphological disease activity will be monitored with SD-OCT (Spectralis HRA + OCT (Heidelberg Engineering, Heidelberg, Germany) or Cirrus (Carl Zeiss Meditec, Inc., Dublin, CA, USA)), colour fundus photography and additionally OCTA-Scans (PLEX Elite 9000, Carl Zeiss Meditec, Inc., Dublin, CA, USA) if available.

Blue-peak blue-laser autofluorescence (BAF), infrared autofluorescence (IRAF), infrared/blue reflectance (IR and BR) and fluorescein angiography (FA) (Spectralis HRA + OCT (Heidelberg Engineering, Heidelberg, Germany), will be performed at baseline and at the end of the study (EOS) visit. Further a quality-of-life questionnaire (QS) will be filled out by all patients at the beginning and end of the study.

At sites where available, patients will receive a microperimetry examination (MP) (MP-3, Nidek Co., Ltd., Japan) and polarization-sensitive optical coherence tomography (PS-OCT, Prototype, available at the Medical University of Vienna, in cooperation with the Department of Medical Physics and Biomedical Engineering) scan at baseline, during the visit in the third month (Visit 3) and at the EOS visit. All activities performed during the visits are summarized in Table 2.

**Table 2:** Schedule of activities.

Assessment/Activity		Study month											
	BSL	Mo 1	Mo 2	Mo 3	Mo 4	Mo 5	Mo 6	Mo 7	Mo 8	Mo 9	Mo 10	Mo 11	Mo 12
BCVA	X	X	X	X	X	X	X	X	X	X	X	X	X
IOP	X	X	X	X	X	X	X	X	X	X	X	X	X
SL	X	X	X	X	X	X	X	X	X	X	X	X	X
FD	X	X	X	X	X	X	X	X	X	X	X	X	X
SD-OCT	X	X	X	X	X	X	X	X	X	X	X	X	X
OCTA	X^a^	X^a^	X^a^	X^a^	X^a^	X^a^	X^a^	X^a^	X^a^	X^a^	X^a^	X^a^	X^a^
CFP	X^a^	X^a^	X^a^	X^a^	X^a^	X^a^	X^a^	X^a^	X^a^	X^a^	X^a^	X^a^	X^a^
BAF	X												X
IRAF	X												X
IR	X												X
BR	X												X
FA	X												X
QS	X												X
PS-OCT	X^a^			X^a^									X^a^
MP	X^a^			X^a^									X^a^
Fluid quantification by AI-algorithm	X^b^	X^b^	X^b^	X^b^	X^b^	X^b^	X^b^	X^b^	X^b^	X^b^	X^b^	X^b^	X^b^
Treatment	X	(X)	(X)	(X)	(X)	(X)	(X)	(X)	(X)	(X)	(X)	(X)	(X)

*Mo* month, *BSL* baseline, *BCVA* best-corrected visual acuity, *IOP* intraocular pressure, *SL* slitlamp biomicroscopy, *FD* fundus biomicroscopy, *SD-OCT* spectral-domain optical coherence tomography, *OCTA* optical coherence tomography-angiography, *CFP* colour fundus photography, *BAF* bluepeak bluelaser autofluorescence, *IRAF* infrared autofluorescence, *IR* infrared reflectance, *BR* Blue reflectance, *FA* fluorescein angiography, *QS* quality-of-life questionnaire, *PS-OCT* polarization-sensitive optical coherence tomography, *MP* microperimetry.

^a^If available at study site.

^b^Only for patients in quantitative study arm (AI-supported PRN).

### Treatment protocols

All eyes will be treated using a PRN regimen with monthly assessments. The anti-VEGF agents applied are determined by each study site according to their routine. Available agents are Ranibizumab (Lucentis^®^, Novartis); Aflibercept (Eylea^®^, Bayer) or Brolucizumab (Beovu^®^, Novartis).

At baseline, study eyes in both arms will receive treatment. Patients in the quantitative (AI-supported) study arm will receive retreatments during Mo1 and/or Mo2 if more than 10 nl of SRF or IRF can be detected in the central 1 mm by an automated artificial intelligence algorithm (AI-based Monitoring Version 1.0, RetInSight, Vienna, Austria). Patients in the qualitative study arm will receive retreatments during Mo1 and/or Mo2 if any fluid in the central 1 mm is detected by the investigator. Afterwards, retreatment will be performed based on quantitative or qualitative disease activity.

SD-OCT scans from patients in quantitative study arm will be analysed by the AI-Software (AI-based Monitoring Version 1.0, RetInSight, Vienna, Austria). Therefore, the SD-OCT volumes, obtained during the monthly visits, will be transferred into the AI database. The AI-based algorithm differentiates and divides specific pixels between the subcategories IRF, SRF, PED and normal tissue. The number of assigned pixels to each fluid type can be computed into an estimation of fluid volumes and their respective location. The amount of time attributed to this process, is dependent on the used volume of OCT data and lies between 1 to 2 min. Pseudonymized image sets are shared by the cloud and are processed by the algorithm on a central server. The clinical investigator will receive the results after completion on a web-based user-interface and a report will be printed out, which will be added to the case report form (CRF).

In case that less than 10 nl SRF or IRF were present during Visit 2 and/or Visit 3 retreatment will only take place, if SRF or IRF increase over the 10 nl threshold. If IRF or SRF volumes in the central millimetre were over the 10 nl threshold during Visit 2 and Visit 3 a retreatment will be triggered by an increase of SRF or IRF over 50% compared to respective fluid volumes during Visit 3.

For patients in the qualitative (conventional) study arm the physician will perform a qualitative fluid assessment, as present or absent, without giving special consideration to the exact volume or change in volume. In the case of fluid presence/detection, retreatment will be necessary according to standard guidelines in the literature [19, 20].

For both arms, a reduction of 5 EDTRS letters or more related to any suspected neovascular activity compared to previous visits and/or new sub-retinal haemorrhage qualifies as a sign of disease activity and fulfils retreatment criteria.

In order to adequately respond to the recent role of IRF fluctuations as harmful for visual acuity [21] and the FLUID study not tolerating high volumes of subretinal fluid [15] we have added a rescue treatment to guarantee best care for the concerned patients in the quantitative study arm. Knowing that a considerably relevant group of patients requires monthly injections [22, 23], patients showing intermittent increase of IRF between the first 2 months, will receive monthly injections until the end of the study to avoid IRF fluctuations. Patients showing signs of an additional substantial increase of SRF while already presenting persistent SRF, will receive bimonthly injections until the end of the study.

In general, the final decision in both treatment arms remains at the discretion of the treating physician. If it is different from what is proposed in the study protocol, the reason will be indicated in the CRF.

### Sample size calculation and statistical analysis

Since our study is the first proof-of-principle study using AI algorithms prospectively, sample size calculation is a difficult task. With regards to the results of the relaxed study arm in the FLUID-Study, we estimate that the mean number of injections in the study arm without AI is at least 8.5. Further we estimated that the standard deviations, in the study arms with and without AI would be 2.6 and 2.25, respectively. We base these estimations on the results obtained by Guymer et al. in the FLUID Study [15].

The significance level (alpha) was set to 0.05. Given a power of 0.8 and a clinically relevant reduction of 10% of the injections during the course of 12 months (total reduction 0.85 injections) we will include 130 patients in each study arm (calculation based on a 2-sided independent t-test). Taking a 10% dropout rate into account our sample size should encompass 145 participants in each study arm, resulting in a total sample size of 290 patients. However, this total sample size was calculated with a rather conservative clinically relevant difference. We expect an even greater difference in total number of injections between the two study arms therefore, an interim analysis is planned after half the patients (145) complete the study in order to verify that our sample size calculations are adequate.

While the mean number of injections will be our primary endpoint, longitudinal changes in BCVA will be considered as a crucial secondary endpoint in order to guarantee treatment effectiveness.

Longitudinal changes in secondary outcomes like quantitative fluid measurement or perfusion of the neovascular lesions, will be investigated for each study arm using a repeated measurement analysis of variance with Geisser-Greenhouse correction for sphericity (ε).

Nominal parameters in the quality-of-life questionnaire will be analysed using a chi²-test. Statistical significance is obtained with a *p* < 0.05.

## Discussion

Currently, best clinical practice proposes that patients with nAMD are treated under the treat and extend (T&E) or PRN regimen based on the presence or absence of IRF and/or SRF according to the physician’s discretion [24]. The proactive T&E regimen is bearing the risk for overtreatment as injections are administered, even if no disease activity is present. The PRN regimen tackles this issue by retreating patients only when disease activity is present. The main drawback for this regimen are the recurrent, burdensome and often monthly reassessment visits [25]. Both of these personalized treatment plans can lead to a significant reduction of injections compared to fixed monthly or bi-monthly regimens, without diminishing the therapeutic benefits [26]. Treating physicians are assessing each of many individual OCT B-Scans, in the form of a macular cube and compare them with evaluations of previous visits in order to detect new or persistent disease activity. Manual quantification of exact fluid volumes in a clinical context is yet not feasible, as OCT grading by professional reading centres requires up to 15 h of annotations per volume [10]. Therefore, most clinical investigators are supported by a pseudo-quantitative metric analysis of CRT provided by the commercially available device set-up. The described conventional process is accompanied by several issues which might explain the differences of treatment success observed between clinical trials and the real-world. Physicians create their own set of rules based on personal experience regarding macular fluid aspects leading to retreatment decisions. This approach is subjective and in some cases retreatment decisions are not fully comprehensible. IRF and SRF volumes are only qualitatively assessed as present or absent, inducing a high risk for overlooking small amounts of macular fluid such as IRF or underestimating the true amount of fluid pooling such as in SRF. Studies showed that a high rate of discrepancies in fluid interpretation has been observed between investigating physicians and reading centres [16]. Foveal IRF and SRF may lead to a measurable excess of central retinal thickness (CRT), but the statistical correlation is low between fluid volumes and CRT particularly in nAMD. Moreover, only a weak correlation between CRT and VA has been established [27]. As CRT does not discriminate between specific compartments of fluid pooling it is an imprecise clinical marker to be used for retreatment decisions [12].

Our study protocol is based on previously substantiated structure-function correlation in exudative processes during nAMD, which has been investigated thoroughly in the recent past [10]. Nonetheless we propose that in order to realize optimal treatment efficacy, all types of pathological fluid fluctuations, whether for IRF or SRF, should be kept to a minimum during long-term care. We decided to use a PRN regimen as the monthly scheduled visits provide granular insights into the dynamics and more specifically into the fluctuations of fluid volumes during anti-VEGF therapy. Recent studies by Chakravarthy et al. suggest that fluctuations of fluid volumes in any compartment during treatment have an unfavourable effect on long-term visual gains [21]. In the FLUID study, AI-based SRF quantification revealed that eyes with an increase in SRF volume experienced consecutive visual loss. Early identification of patient subgroups with alternating cycles of lesion activity and quiescence will be easier and more comprehensive using our proposed AI-algorithm.

A study conducted by Michl et al., using automated fluid quantification indicated that IRF volumes diminished by over 90% between the baseline visit and the first month. SRF volumes were even reduced by over 95% [28]. This suggests that little to no fluid volumes might be detectable in most patients after the first few injections. Accounting for these assumptions we decided to omit the conventional loading dose in our study protocol. The consistently low residual volumes in IRF and SRF which we found in precedent fluid analyses in clinical trials such as HARBOR and HAWK&HARRIER [29, 30] were used to define the lower threshold for retreatment indication.

A quantitative measurement of fluid by an objective AI-algorithm outmanoeuvres most of the obstacles present in qualitative fluid assessment. Keenan et al. suggested that an AI-algorithm could surpass retinal specialists in accurately detecting fluid pooling, especially IRF and in difficult cases with lower fluid volumes in fewer B-scans [31]. AI-algorithms can analyse large volumes of OCT-imaging data in a fraction of the time compared to what a human investigator would need for the same task [32]. Additionally, all patients would be retreated using the same comprehensible and at the same time personalized treatment criteria. Retreating with a special focus on reducing IRF and SRF volumes might lead to non-inferior gains in visual acuity despite a reduced number of injections. A number of publications have underlined these assumptions and suggested to include AI-based fluid assessment in prospective studies as an additional tool for retreatment decisions [29]. Even with multiple retrospective analyses using advanced analytical tools, insights of fluid recurrence and retreatment patterns are limited to what has been done by protocol and not necessarily what would have been an ideal retreatment strategy based on fluid/function correlation. Only prospective studies can identify optimized retreatment schemes for the future which are based on a realistic fluid volume management. This is a necessary step towards the inclusion of AI into everyday clinical practice and should be considered a “private” reading centre at hands in every doctor’s office. Furthermore, its use might extend beyond nAMD to other macular diseases.

With regards to the developments during the Covid-19 pandemic, numerous healthcare systems have sought to minimize face-to-face contacts between patients and treating physicians. The concept of a “virtual” medical retina clinic has been brought up as a promising and innovative approach [33]. In certain proposed models follow-up visits for nAMD patients would consist of a VA-test and an OCT scan, omitting a physical and direct assessment of the retina by fundoscopy [34]. These new therapy models showed promising results with an increased cost efficiency for clinics [35]. Yet, this approach comes with a high risk for misinterpretation as has been shown by the Moorfields group [36]. Combining these concepts with AI-Algorithms like the one we propose will diminish the risk for undertreatment while adhering to the principal of social-distancing and reducing the mean time for outpatient appointments. Moreover, such automated precision measurements can be offered in the vicinity of the patients by any OCT user.

## Summary

### What was known before

- Real-world outcomes are disappointing in comparison to visual acuity gains in clinical trials.
- Automated artificial intelligence (AI)-algorithms have shown robust results for fluid quantification in retrospective analysis in neovascular age-related macular degenerations (nAMD).
- Using AI it has become possible to provide real-time quantifications of macular fluid for more precise retreatment decisions.

### What this study adds

- Automated AI-algorithms are for the first time implemented into real-world clinical practice.
- A prospective retreatment protocol based on the objective assessment of fluid activity instead of presence alone will introduce novel standards for optimized retreatment over long-term follow-up.
- Optimized treatment regimen will increase the treatment capacity of healthcare systems and therefore reduce vision loss in a real-world setting.
